# Design Space and Control Strategy for the Manufacturing of Wet Media Milled Drug Nanocrystal Suspensions by Adopting Mechanistic Process Modeling

**DOI:** 10.3390/pharmaceutics16030328

**Published:** 2024-02-26

**Authors:** André Bitterlich, Andrej Mihorko, Michael Juhnke

**Affiliations:** 1 Novartis Pharma AG, Fabrikstrasse 2, 4056 Basel, Switzerland; 2 Novartis Pharmaceutical Manufacturing LLC, Verovškova Uulica 57, 1000 Ljubljana, Slovenia; andrej.mihorko@novartis.com; 3 F. Hoffmann-La Roche Ltd., Grenzacherstrasse 124, 4070 Basel, Switzerland; michael.juhnke@roche.com

**Keywords:** design space, control strategy, quality-by-design, risk assessment, mechanistic, modeling, wet stirred media milling, nanocrystal, nanosuspension, drug nanoparticles

## Abstract

Wet media milling is a fully industrialized technology for the manufacturing of drug nanocrystal suspensions. This work describes the development of an advanced control strategy and an associated design space for a manufacturing process at a commercial scale. Full-scale experiments and mechanistic process modeling have been used to establish a physically reasonable control strategy of factors relevant to the quality attributes of the nanocrystal suspension. The design space has been developed based on a mature mechanistic process model of the wet media milling procedure. It presents the process–product attribute relationship between a multidimensional range of measured process parameters and a range of the product-quality attribute mean particle sizes. The control strategy allows for simple, robust, and sound scientific process control as well as the operational flexibility of the suspension batch size. This is an industrial case study of control strategy and design-space definition with the crucial contribution of mechanistic process modeling for an intended commercial manufacturing process.

## 1. Introduction

Over the last three decades, nanocrystals have emerged as a formulation strategy to improve bioavailability-related problems of poorly soluble drugs, enhance clinical convenience, and enable specific therapeutic benefits [1,2,3,4,5,6,7]. Until now, several commercialized nanocrystalline drug products have reached the market for oral, ocular, subcutaneous, intramuscular and intravenous routes of administration [8,9]. The manufacturing of drug nanocrystals for industrial applications can be accomplished by wet stirred media milling technology [9,10,11,12,13,14,15]. In essence, a size reduction of drug particles takes place in an aqueous suspension in the presence of stabilizers between colliding grinding media. The technology is considered a versatile drug-delivery platform, owing to its industrial applicability for oral, ocular, and injectable products from the pre-clinical to the commercial stage.

The design space and control strategy of a manufacturing process are firmly anchored in the concept of quality-by-design (QbD). QbD has been the focus within the last two decades, to advance development and emphasize product and process understanding and process control based on sound science and quality risk management. The fundamental principles are described in the quality guidelines of the International Conference on Harmonization of Technical Requirements for Registration of Pharmaceuticals for Human Use (ICH) Q8, Q9 and Q10 [16,17,18,19]. In addition, ICH Q11 [20] and ICH Q12 [21] have been elaborated to facilitate the application for marketing authorization and lifecycle management, including the identification of critical quality attributes, development of experimental design space and control strategies for quality assurance, and post-approval change management [20,21]. Up-to-date overviews of the concept of QbD, current status, application, challenges, and future perspectives can be found elsewhere [22,23,24,25].

The risk assessment in QbD systematizes the product and process understanding generated during development to define a robust process, operated within a design space or parameter ranges and associated control strategy, to ensure the manufacturing and control of acceptable product quality [22,23,24,25,26]. In general, input material attributes, equipment parameters and scale, process parameters and batch size, and the holding period of intermediate products are considered for the individual unit operations selected for product manufacturing. Nowadays, a statistical design of experiment (DoE) is usually applied to investigate the relationship and impact of the input material attributes and parameters on potential critical quality attributes (pCQAs), and finally, to define the critical material attributes (CMAs), critical process parameters (CPPs), and related ranges for the manufacturing of the acceptable product quality of the critical quality attributes (CQAs). The CQAs and their acceptable ranges interlink product quality with clinical safety and efficacy in the quality target product profile (QTPP) [17]. The control strategy is derived from the outcome of the development effort and a risk assessment, taking into consideration CMAs, CPPs, and CQAs. The control strategy may consider in-process quality control measurements using at-line/off-line analytical methods or in-line/on-line process analytical technologies.

Process modeling has received increasing attention in recent years within the framework of QbD, particularly driven by the modernization efforts of the pharmaceutical industry [27,28]. From a scientific point of view, process models can be classified into empirical, semi-empirical, and mechanistic (first principles) models. Empirical models include multivariate statistical analysis approaches. Mechanistic process models are considered most advantageous because they have physical rationales; include inputs and outputs; and most importantly, the internal state of the system. Furthermore, it is open for scalability. However, mechanistic process models are rarely developed for pharmaceutical processes due to the time and resources needed for development, including the requirements for modeling expertise and the extent of resources necessary for model validation [28]. This is particularly the case for pharmaceutical processes, wherein particles, granules, powders, etc., are involved and, therefore, complex models need to be developed and validated, e.g., population balance models, finite element models, computational fluid dynamic models, discrete element models, and coupled models thereof [29,30,31,32]. Process models can be developed and applied for different purposes, e.g., process design, development, optimization, scale-up, identification of CMAs and CPPs, risk evaluation, and material traceability. Most advanced process models can be incorporated into the description of the design space and as a component in the control strategy for either process monitoring or active process control to ensure the acceptable product quality of the manufacturing process [27]. Regulatory guiding principles for process model development, implementation, documentation, validation, and verification during the lifecycle are described in the related quality guidelines [19]. In general, process models are categorized according to their contribution to ensuring product quality with different regulatory implications:

- Low impact: Models used to support product and/or process development.
- Medium impact: Models used for product quality assurance, but in combination with other indicators of quality.
- High impact: Models whose predictions are the main indicators of product quality.

The comprehensive QbD approach for wet stirred media milling processes is described by different authors in accordance with the above overview [33,34,35]. Essentially, the complex manufacturing process for nanocrystal suspensions is often developed using DoEs to identify CMAs and CPPs. For instance, Jog and Burgess [33] identified API concentration, polymer concentration, and surfactant concentration as CMAs, and agitator speed, pump speed, and milling time as CPPs. It is well known that CMAs and CPPs can be interconnected in relation to their impact on CQAs. The design space is most often defined based on the statistical analysis of the experimental results and the acceptable ranges of the CQAs. There is only limited information reported on the control strategy of wet stirred media milling processes. However, the attributes, parameters, and quality control measurements to take into consideration for the control strategy can be grouped as follows:

- Material attributes of input components, e.g., drug particle size, stabilizer quality.
- Equipment parameters, e.g., agitator material, mesh size of sieve cartridge.
- Set process parameters, e.g., agitator shaft speed, pump speed, cooling-liquid temperature into grinding chamber jacket.
- Resulting actual process parameters, e.g., nanocrystal suspension temperature.
- Resulting cumulative process parameters, e.g., milling time.
- In-process quality control measurements, e.g., nanocrystal particle size.

Many of the material attributes, set process parameters, and resulting actual and evolving process parameters necessarily require a proven acceptable range to cover, at minimum, the related true values occurring during repetitive batch manufacturing. The in-process control measurements necessarily require a range of acceptable quality. Chen et al. [8] discussed the complex task from the point of a regulator body and highlighted the inadequate control of the process using only the cumulative process parameter number of turnover cycles.

The purpose of this work is to present an advanced design space and control strategy for a wet stirred media milling process for the manufacturing of a nanocrystal suspension by adopting mechanistic process modeling. The mechanistic process models are based on a stress-energy model developed by Kwade more than 20 years ago [36,37,38,39,40] and a simple energy balance of the suspension in the grinding chamber. The mechanistic process models were experimentally established on a commercial equipment scale. Finally, an exemplary design space with a corresponding control strategy is presented for the manufacturing of nanocrystal suspensions using a medium-impact model approach for CQA mean particle size.

## 2. Materials and Methods

### 2.1. Materials

A proprietary crystalline drug provided by Novartis Pharma AG was used for this study. The drug compound is a weak base showing high permeability and low solubility at physiological pH, with a molecular weight above 500 g/mol, a melting point above 180 °C, and water solubility below 0.1 mg/mL. A typical polymer and surfactant are used for the stabilization [3,11,12,13,14,15], and purified water was the liquid continuous medium for the nanocrystal suspension.

The drug and stabilizer compositions were constant throughout the experiments, with drug concentration above 20% *w*/*w* and small stabilizer concentration below 5% *w*/*w* in purified water.

### 2.2. Experimental Methods

**Figure 1:**
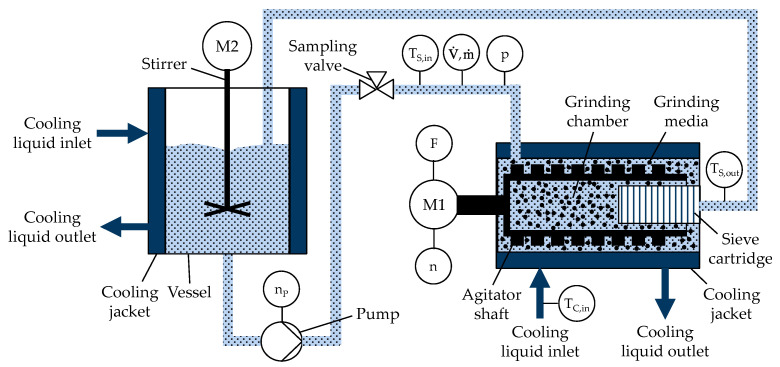
Schematic drawing of milling setup including (a) Components of wet stirred media mill: grinding chamber, grinding media, sieve cartridge, cooling jacket, agitator shaft and motor drive (M1), (b) Components of recirculation vessel: vessel, cooling jacket, stirrer and motor drive (M2), (c) Pump, (d) Sampling valve, and (e) Relevant sensors: Suspension inlet temperature into grinding chamber (T_S,in_), suspension outlet temperature from grinding chamber (T_S,out_), cooling-liquid inlet temperature into grinding chamber jacket (T_C,in_), suspension inlet pressure into grinding chamber (p), suspension volume flow ($\dot{V}$) and mass flow (ṁ), number of revolutions of agitator shaft (n), number of revolutions of pump (n_P_), load at agitator shaft (F).

Experiments were conducted on a commercially available wet stirred media mill (Netzsch Feinmahltechnik GmbH, Selb, Germany, DeltaVita 10000) in recirculation mode. It was equipped with a peristaltic pump, which facilitated the transportation of the suspension from the recirculation vessel through the grinding chamber and back into the vessel. A schematic drawing of the experimental setup including the relevant sensors is depicted in Figure 1.

The mill design was improved with a load cell to enable the compliant measurement of the power consumption of the agitator shaft. The load cell regularly allows calibration to ensure quality assurance. The power at the agitator shaft of the mill (P) is calculated according to Equation (1) with torque at the agitator shaft (T_Agi_) and angular frequency of the agitator shaft (ω), considering the load measured at the load cell sensor (F), the constant radius of the load cell sensor to the longitudinal axis of the agitator shaft (r), and the number of revolutions of the agitator shaft (n).

(1) $$P=T_{Agi}\cdot \omega =F\cdot r\cdot 2\cdot \pi \cdot n$$

The inlet temperature (T_S,in_) and outlet temperature (T_S,out_) of the suspension were measured with thermocouple elements. The grinding chamber of the mill and the recirculation vessel were both equipped with a double jacket to allow for the cooling of the suspension. Cooling-liquid flow rates and cooling-liquid inlet temperatures for the grinding chamber (T_C,in_) and vessel (T) were controlled by separate cooling systems. The stirrer speed of the recirculation vessel was adapted according to the vessel size and batch size for the specific experiment. Experiments were conducted with low set volume flow of 5 L/min at the beginning, followed by a ramp up during processing after three batch turnover cycles, to the selected set volume flow of the suspension. Further processing was performed with a constant volume flow of the suspension until a pre-defined end point of the mass specific energy. Prior to each experiment, a no-load power measurement was conducted to calculate the net power input. The mass specific energy and no-load power measurement are described in detail in Section 2.4.1. Samples were taken during processing by a three-way valve introduced into the pipe between the pump and grinding chamber. The valve allows for representative sampling of the suspension during processing from the pipe feed into the grinding chamber.

### 2.3. Analytical Methods

Particle size was characterized for the input crystal suspension by laser light diffraction (LLD) and for the wet media milled nanocrystal suspension by photon correlation spectroscopy (PCS). LLD analysis was performed using the equipment model Sucell/Helos, Sympatec GmbH, Clausthal-Zellerfeld, Germany. The obtained scattered light intensities were evaluated according to the Fraunhofer diffraction model and reported by the volume of the 90th percentile of the cumulative undersize distribution (x_90,3_). PCS analysis was performed using the equipment model Zetasizer Nano ZS, Malvern Panalytical Ltd., Worcestershire, UK. The obtained scattered light intensity dynamics were evaluated according to the cumulant method and results were reported by the scattering intensity weighted mean particle size (x_PCS_) and the polydispersity index (PdI). Samples for LLD and PCS analysis were diluted with purified water for an adequate obscuration level and scattering intensity, retrospectively. The PCS method was validated and demonstrated an intermediate precision for x_PCS_ of 0.9% RSD and for PdI of 7.4% RSD.

### 2.4. Process Models

#### 2.4.1. Mechanistic Process Model

The stress-energy model developed by Kwade [36,37,38,39,40] is a mechanistic process model that is currently fully mature, well understood, and successfully applied for different applications in industry and academia, e.g., for mining [41], battery [42,43,44], pigment [45,46], ceramic [47], and pharmaceutical [10,48,49] applications. The stress-energy model describes the impact of equipment and process parameters on particle size for wet stirred media milling processes according to the following equation:

(2) $$E_{M}=SE\cdot SN$$

where E_M_ is the mass specific energy, SE is the stress energy of the grinding media, and SN is the total stress number. The mass specific energy E_M_ can be calculated according to the following discrete equation,

(3) $$E_{M,i}=\sum_{i=0}^{i=j}E_{M,i-1}+\frac{\left(P_{i}-P_{0,i}\right)\cdot \left(t_{i}-t_{i-1}\right)}{M}$$

where i represents the index of summation, i = 0 is the lower bound of summation representing the index at the start of milling duration, i = j is the upper bound of summation representing the index at total milling duration, E_M,i−1_ is the mass specific energy calculated at milling duration with index i − 1, P_i_ is the power draw of the agitator shaft of the wet media mill at milling duration with index i, P_0,i_ is the no-load power draw of the agitator shaft of the wet media mill at milling duration with index i, M is the total mass of the suspension, t_i_ is the milling duration at index i and t_i−1_ is the milling duration at index i − 1. The average stress energy $\bar{SE}$ can be calculated by the following equation:

(4) $$\bar{SE}=\rho_{GM}\cdot d_{GM}^{3}\cdot \frac{1}{j}\cdot \sum_{i=0}^{i=j}v_{t,i}^{2}$$

where ρ_GM_ is the density of the grinding media, d_GM_ is the diameter of the grinding media, and $v_{t,i}$ is the tip speed of the agitator shaft of the wet media mill at milling duration with index i. The stress number, SN, is proportional to the following equation for wet media mills operated in recirculation mode:

(5) $$SN\propto n\cdot N_{GM}\cdot T$$

where n is the speed of the agitator shaft of the wet media mill, N_GM_ is the number of grinding media in the grinding chamber, and Τ is the ideal residence time of the suspension in the grinding chamber. The parameter N_GM_ is constant for the manufactured batches. Parameter n is approximately constant within an experiment. The targeted variation of parameter n is expected to have a negligible impact within the investigated range of this study. Therefore, the stress number, SN, is directly related to the ideal residence time, which is proportional to the number of turnover cycles (N). The parameter number of turnover cycles (N) can be calculated according to the following discrete equation:

(6) $$N_{i}=\sum_{i=0}^{i=j}\frac{m_{i-1}+{\dot{m}}_{i}\cdot \left(t_{i}-t_{i-1}\right)}{M}$$

where m_i−1_ is the cumulative mass of the suspension transported through the grinding chamber at milling duration with index i, ṁ_i_ is the suspension mass flow at milling duration with index i, M is the total mass of the suspension, t_i_ is the milling duration at index i, and t_i−1_ is the milling duration at index i − 1.

#### 2.4.2. Energy Balance

**Figure 2:**
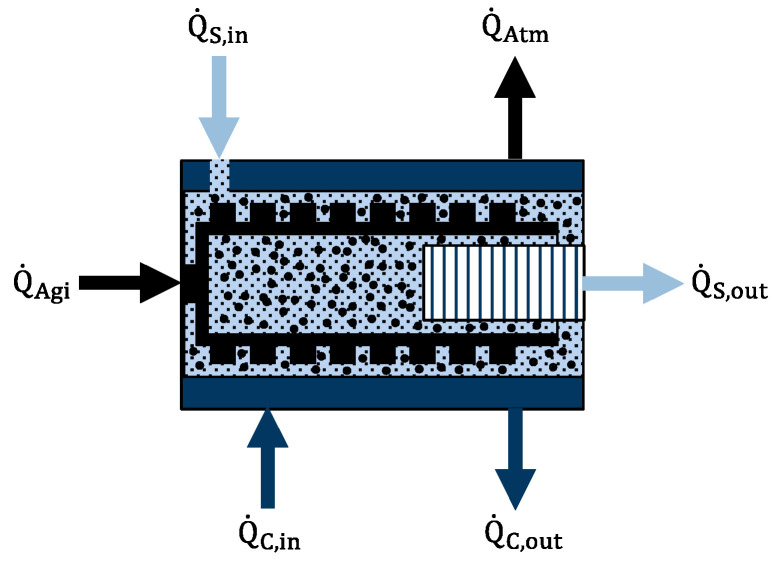
Schematic drawing of the energy balance for the wet media milled suspension in the grinding chamber, including input heat of the suspension $\left({\dot{Q}}_{S,in}\right)$, energy introduced by the mechanical agitation of the agitator shaft and associated grinding media ${\dot{(Q}}_{Agi}$), input heat of the cooling liquid (${\dot{Q}}_{C,in}$), output heat of the suspension $({\dot{Q}}_{S,out}$), output heat of the cooling liquid ${\dot{(Q}}_{C,out}$) and loss of heat to the outer atmosphere (${\dot{Q}}_{Atm}$).

The impact of equipment and process parameters on the energy introduced into the suspension in the grinding chamber—the temperature increase in the suspension in the grinding chamber—can be derived by an energy balance according to Figure 2.

The general energy balance of the suspension in the grinding chamber can be derived with the following equation:

(7) $${\dot{Q}}_{S,out}-{\dot{Q}}_{S,in}={\dot{Q}}_{C,in}-{\dot{Q}}_{C,out}+{\dot{Q}}_{Agi}-{\dot{Q}}_{Atm}$$

where further contributing factors are neglected, e.g., heat generated at the bearings of the agitator shaft. The heat of the suspension can be calculated according to the following equation:

(8) $${\dot{Q}}_{S,out}-{\dot{Q}}_{S,in}={\dot{m}}_{i}\cdot c_{p,S}\cdot \left(T_{S,out,i}-T_{S,in,i}\right)$$

where ${\dot{m}}_{i}$ is the mass flow of the suspension through the grinding chamber at milling duration with index i, $c_{p,S}$ is the heat capacity of the suspension, T_S,in,i_ is the inlet temperature of the suspension flowing into the grinding chamber, and T_S,out,i_ is the outlet temperature of the suspension flowing out of the grinding chamber at milling duration with index i. The input energy of the agitator shaft can be calculated according to the following equation:

(9) $${\dot{Q}}_{Agi}=P_{i}-P_{0,i}$$

where P_i_ is the power draw of the agitator shaft of the wet media mill at milling duration with index i, P_0,i_ is the no-load power draw of the rotor of the wet media mill at milling duration with index i. The heat flow of the cooling liquid can be calculated according to the following equation:

(10) $${\dot{Q}}_{C,out}-{\dot{Q}}_{C,in}={\dot{m}}_{C,i}\cdot c_{p,C}\cdot \left(T_{C,out,i}-T_{C,in,i}\right)$$

where ${\dot{m}}_{C,i}$ is the mass flow of the cooling liquid through the cooling installation of the grinding chamber at milling duration with index i, $c_{p,C}$ is the heat capacity of the cooling liquid, T_C,in,i_ is the inlet temperature of the cooling liquid flowing into the jacket of the grinding chamber and T_C,out,i_ is the outlet temperature of the cooling liquid flowing out of the jacket of the grinding chamber at milling duration with index i. The loss of heat flow to the outer atmosphere can be calculated according to the following equation:

(11) $${\dot{Q}}_{Atm}=\alpha \cdot A\cdot \left(T_{Atm}-T_{S}\right)$$

where α is the heat transfer coefficient between the outer surface of the grinding chamber and the outer air atmosphere, A is the area of the outer surface of the grinding chamber, T_Atm_ is the temperature of the outer air atmosphere, and T_S_ is the temperature of the outer surface of the grinding chamber. The individual terms can be consolidated according to the general energy balance and converted according to the outlet temperature of the suspension from the grinding chamber at milling duration with index i into the following equation:

(12) $$T_{S,out,i}=\frac{{\dot{m}}_{C,i}\cdot c_{p,C}\cdot \left(T_{C,in,i}-T_{C,out,i}\right)+{\dot{Q}}_{Agi,i}-{\dot{Q}}_{Atm}}{{\dot{m}}_{i}\cdot c_{p,S}}+T_{S,in,i}$$

Therefore, the outlet temperature of the suspension from the grinding chamber at milling duration with index i (T_S,out,i_) is a representative process parameter for controlling the relevant process parameters for the energy input into the suspension in the grinding chamber. For the sake of convenience, the average outlet temperature of the suspension from the grinding chamber can also be calculated for a manufactured batch (${\bar{T}}_{S,out}$).

## 3. Results and Discussion

### 3.1. Experimental Design

The nanocrystal suspension is an intermediate product and further processed into a solid oral dosage form. A QbD-based systematic risk assessment was used for the wet stirred media milling process to identify input material attributes, environment, equipment parameters, process parameters, and CQAs. Drug substance properties, general scientific knowledge, and prior knowledge gained during formulation, process, and product development were considered to ensure the QTPP of the drug product. Supporting experimental investigations were performed at a laboratory/pilot scale for development, considering:

- Material attributes, including input drug substance; the stabilizer and water of the suspension and their formulation composition; and the liquid for the mechanical seal.
- Equipment parameters, including the agitator shaft material, grinding chamber liner material, and sieve cartridge mesh size.
- Process parameters, including grinding media material, diameter and fill level in the grinding chamber, agitator shaft speed, suspension volume flow, grinding chamber cooling-liquid volume flow and temperature, suspension inlet temperature into the grinding chamber, and suspension outlet temperature from the grinding chamber.
- Potential critical quality attributes of the nanocrystal suspensions relevant for down-streaming and the final drug product, e.g., assay, solid form, impurities, particle size distribution, pH, density, zeta-potential, viscosity, wear from grinding media, lining and agitator shaft in chemical composition and quantity.

Experimental approaches for the development of the material attributes, equipment and process parameters, and potential critical quality attributes (pCQAs) are reported in the literature [33,34,35]. Furthermore, different engineering approaches for the scale-up of the wet stirred media milling process from a laboratory/pilot to a commercial scale are available [10,40,49,50]. For this study, the established risk assessment; supporting experimental investigations for formulation, process, and product development at the laboratory/pilot scale; and the scale-up to commercial scale are not presented.

The development of the design space and control strategy of the nanocrystal-suspension manufacturing process at the commercial manufacturing scale is presented hereinafter. Experiments were conducted in the commercial manufacturing setup, taking into consideration the following aspects:

- Experimental results from formulation and process development at the laboratory/pilot scale.
- Mechanistic process models for the wet media milling process and the energy balance of the suspension in the grinding chamber (see Section 2.4.1 and Section 2.4.2).
- Established risk assessment for the experimental setup at a commercial scale.
- Process efficiency, flexibility, simplicity, robustness, and integration within the drug-product commercial manufacturing process.
- Demands and constraints of the entire process of drug-substance and drug-product development and clinical supplies.

Table 1 provides a general overview of the defined and investigated attributes and parameters for the experimental design at a commercial scale. The previously mentioned aspects with the necessary depth were considered to ensure an anticipatory risk assessment of the commercial manufacturing process. pCQAs were identified based on the formulation and process development at the laboratory/pilot scale, the QTPP for the drug product, and the risk assessment for the commercial manufacturing setup.

### 3.2. Experimental Results

The conducted experiments including the investigated parameters and obtained results are summarized in Table 2. Additional samples were systematically removed during processing for experiments No. 1–12 and analyzed for mean particle size and polydispersity index.

**Figure 3:**
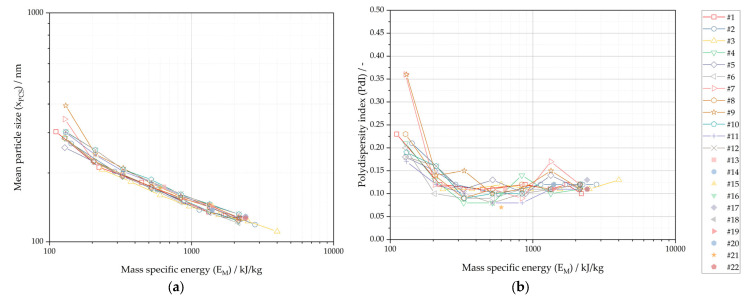
Experimental results of nanocrystal suspension plotted against mass specific energy (E_M_): (**a**) Mean particle size (x_PCS_); (**b**) Polydispersity index (PdI).

Figure 3 presents the experimental results for mean particle size and polydispersity index plotted against mass specific energy. The size reduction of the drug compound during processing increases with increasing mass specific energy. The plotted process–product attribute relationship is well correlated within the investigated parameter ranges. This is an expected outcome for the mechanistic process model and in good agreement with the literature [40,48,49]. The relative standard deviation (RSD) of mean particle size at a constant mass specific energy is below 3.5% RSD for x_PCS_ below 180 nm. This demonstrates the excellent correlation of mean particle size with mass specific energy and the high reproducibility of the process within the investigated parameter ranges. It also supports the assumption that the variation in the speed of the agitator shaft of the wet media is negligible within the investigated range (see Section 2.4.1.). The polydispersity index initially decreases with increasing mass specific energy and is constant with a PdI of about 0.11 ± 0.03 for specific energies above 200 kJ/kg. Therefore, the polydispersity index is well correlated for processes operated at higher specific energies.

Apparently, input drug substance particle size (x_90,3_) showed no effect on mean particle size and the polydispersity index within the investigated range of x_90,3_ of 4.2 to 7.6 μm. The suspension outlet temperature from the grinding chamber (${\bar{T}}_{S,out}$) showed a slight increase with an increase in agitator shaft speed and a decrease in ${\bar{T}}_{S,out}$ with an increase in suspension volume flow, in line with Equation (12). However, the temperature difference of the suspension at the outlet of the grinding chamber is small when comparing all experiments (${\Delta \bar{T}}_{S,out}$ = 9 °C), and no effect could be identified on mean particle size and the polydispersity index within the investigated range.

**Figure 4:**
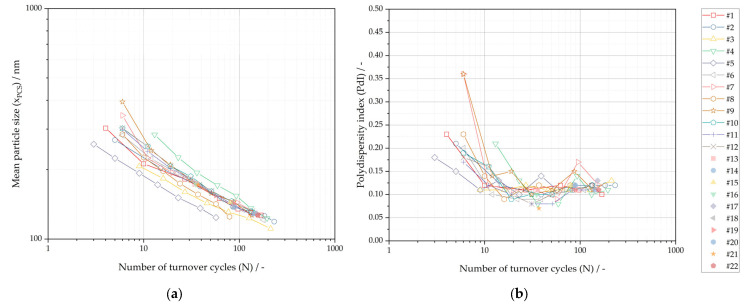
Experimental results of nanocrystal suspension plotted against number of turnover cycles (N): (**a**) Mean particle size (x_PCS_); (**b**) Polydispersity index (PdI).

The mean particle size and the polydispersity index are plotted against the number of turnover cycles in Figure 4. The mean particle size at a constant number of turnover cycles illustrates a high scatter, whereas the polydispersity index shows a similar correlation for above 40 turnover cycles compared to process parameter mass specific energy. The relative standard deviation of mean particle size at a constant number of turnover cycles is below about 18% RSD for x_PCS_ below 200 nm. The poor correlation accuracy is in good agreement with the limitations highlighted by Chen et al. [8] for the sole control of the size-reduction process by the number of turnover cycles.

**Figure 5:**
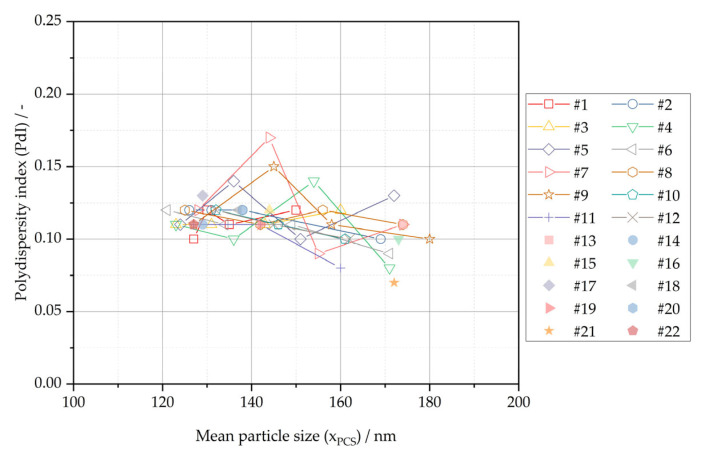
Relationship of mean particle size (x_PCS_) and polydispersity index (PdI) in nanocrystal suspension between x_PCS_ of 120 nm and 180 nm.

The quality attributes of mean particle size and the polydispersity index originate from the identical analytical method and sample measurement. The relationship of both quality attributes is plotted for mean particle sizes between 120 and 180 nm in Figure 5.

There is possibly a minor negative correlation between the polydispersity index and mean particle size in the plotted range. However, as an integrated whole, the relative standard deviation of the polydispersity index is about 15% RSD within the plotted range. This is considered a negligible deviation, in view of the intermediate precision of the analytical method for the polydispersity index of 7.4% RSD—see Section 2.3. Therefore, mean particle size is the sole relevant critical quality attribute for the evaluation of the size distribution of the nanocrystal suspension, and the polydispersity index can be neglected for mean particle sizes between 120 and 180 nm.

Additional samples were removed from the recirculation vessel during processing for selected experiments for comparison with samples removed from the sampling valve. Samples from the vessel were removed with a glass pipette. The comparison revealed an adequate comparison between samples from the vessel and the valve for mean particle size and the polydispersity index within the intermediate precision of the analytical method (see Section 2.4.), for the manufacturing with more than 10 turnover cycles.

### 3.3. Design Space

A design space is defined as “The multidimensional combination and interaction of input variables (e.g., material attributes) and process parameters that have been demonstrated to provide assurance of quality” according to ICH Q8 [17]. The guideline provides recommendations for single-unit operation vs. multiple-unit operations design spaces; the relationship to equipment scale; and examples for the development and presentation in a regulatory submission. The concept of design space is an optional QbD element. Interestingly, design spaces are not often submitted for regulatory submission, e.g., on average, 8% of submissions between 2009 and 2023 in EMEA [25] and 2% of submissions between 2009 and 2018 in Japan included a design space [51]. Misunderstandings of the design space are critically discussed from an industrial perspective by Watson et al. [52]. This includes the misconception of the requirement of multidimensional DoE for the development of the design space. DoE is often the method of choice for the investigation of multidimensional interactions of input material attributes, process parameters, and CQAs due to scientific complex relationships. However, prior scientific knowledge, including a mechanistic understanding of materials science and processes, may result in a simpler approach to the development of a design space [52]. 

The design space herein developed for this unit operation and equipment scale takes into consideration the following aspects:

- Experimental results at commercial manufacturing setup—see Section 3.2.
- Mechanistic process models for the wet media milling process and the energy balance of the suspension in the grinding chamber—see Section 2.4.1 and Section 2.4.2.
- Established CQAs for the nanocrystal suspension and the final drug product.
- Updated risk assessment for the commercial manufacturing process.

Mean particle size (x_PCS_) was identified as a CQA for the nanocrystal suspension (see Section 3.1 and Section 3.2). The relationship between nanocrystal mean particle size and final drug-product CQAs for release and stability and the linkage to biopharmaceutical performance according to ICH Q6A [53] are not presented for this study. In the further course of this study, an exemplarily range of the acceptable criteria of 120 nm ≤ x_PCS_ ≤ 180 nm was assumed. 

The multidimensional interaction of the process–product attribute relationship is described by the mechanistic process model according to Kwade [36,37,38,39,40]—see Equation (2). The design space is basically constructed according to Equation (2), with mass specific energy (E_M_) and number of turnover cycles (N) as representative cumulative process parameters for the total stress number (SN). Therefore, mass specific energy (E_M_) and the number of turnover cycles (N) were designated as CPPs. Equation (2) is solved by these two parameters, and also, the process–product attribute relationship is fully controlled. Consequently, average stress energy ($\bar{SE}$), as a representative cumulative process parameter for stress energy (SE), is designated as non-CPP, albeit being restricted to the experimentally justified acceptable parameter range. The process-parameter suspension outlet temperature from the grinding chamber (${\bar{T}}_{S,out}$) showed no impact on the quality attributes of the nanocrystal suspension. Therefore, the suspension outlet temperature from the grinding chamber is designated as non-CPP and restricted to the experimentally justified acceptable parameter range.

**Figure 6:**
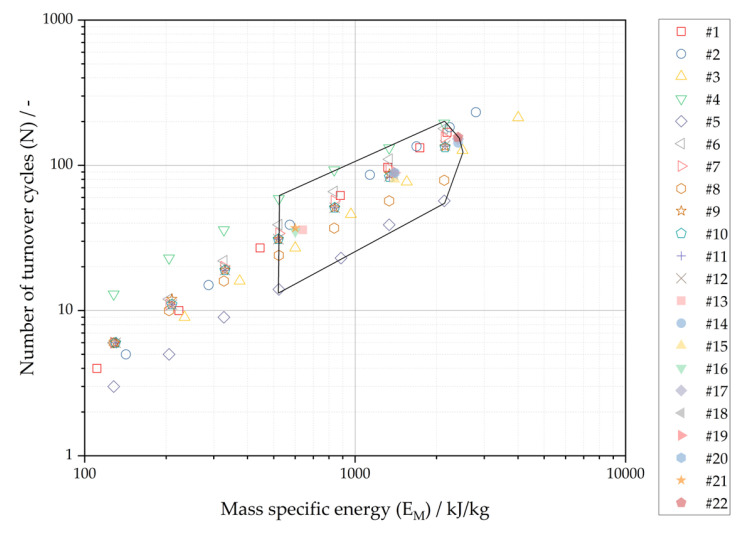
Experimental results and design space (hexagon) of resulting cumulative process parameters’ mass specific energy (E_M_) and number of turnover cycles (N) for the nanocrystal suspension manufacturing process with an exemplary range of the acceptance criteria mean particle size (x_PCS_) of 120 nm ≤ x_PCS_ ≤ 180 nm.

**Figure 7:**
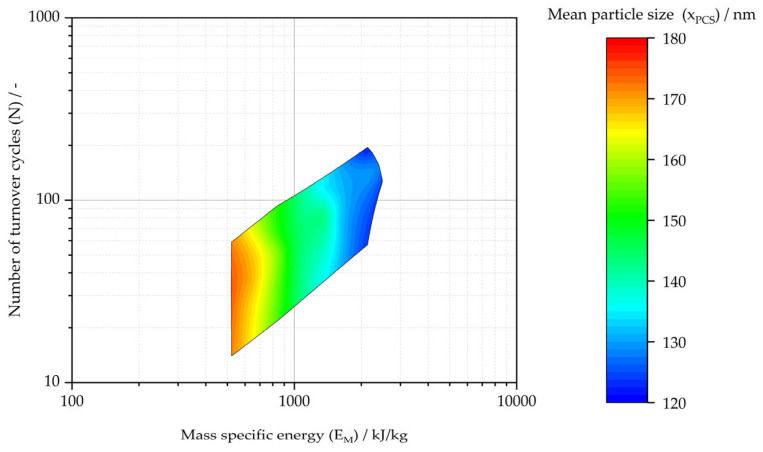
Design space with interpolated color gradient for mean particle size of resulting cumulative process parameters’ mass specific energy (E_M_) and number of turnover cycles (N) for the nanocrystal suspension manufacturing process with an exemplary range of the acceptance criteria mean particle size (x_PCS_) of 120 nm ≤ x_PCS_ ≤ 180 nm.

The experimental results are plotted in Figure 6 by the cumulative process parameters number of turnover cycles (N) against mass specific energy (E_M_). The hexagon shown in Figure 6 highlights the design space for the exemplary range of the acceptance criteria for mean particle sizes of 120 nm ≤ x_PCS_ ≤ 180 nm. Figure 7 illustrates the multidimensional design space. The interpolated color gradient illustrates the relationship of the measured cumulative process parameters (N vs. E_M_) and product attributes (x_PCS_) within the design space.

### 3.4. Control Strategy

A control strategy is defined as “A planned set of controls, derived from current product and process understanding that ensures process performance and product quality” according to ICH Q10 [16]. It is the risk-based link between a product’s control and patient needs and presents the state-of-the-art industry’s approach to product quality assurance. The control strategy is considered the most important QbD element [52].

Table 3 presents the control strategy herein developed in accordance with the experimental findings above (see Section 3.1 and Section 3.2), the defined design space (see Section 3.3), and the updated risk assessment for the commercial manufacturing process. The critical designation of attributes and parameters is defined and individual control measures are indicated, including certificate of analysis (CoA); industrial process control system (IPCS); master batch record related procedures and manufacturing execution system (MBR/MES); and in-process quality control (IPC). Acceptable ranges for input material attributes and process parameters are given where appropriate, and the acceptance criteria for the in-process control-attribute mean particle size are specified.

A medium-impact model is utilized for the control strategy and associated design space. CPPs are defined according to their impact on CQA mean particle size. Related acceptable ranges were experimentally justified in the commercial manufacturing setup. Non-critical material attributes and process parameters are controlled by appropriate control measures, including CoA, MBR/MES, and IPCS. Acceptable ranges were defined according to their proven ranges for the commercial manufacturing setup. These control measures already allow a high level of quality assurance during repetitive batch manufacturing. Potential failure modes like operational failure during the weighing of the suspension batch size (M), operational failure of the determination of the no-load power draw (P_0_), and data failure of the mass flow sensor during processing (ṁ_i_) remain low-level risks for deviations. Therefore, IPC testing the CQA mean particle size (x_PCS_) after batch manufacturing was introduced into the control strategy based on a risk–cost–benefit analysis.

The control strategy represents a Level 2 control strategy according to Yu et al. [26]. Individual input material attributes, equipment, and set process parameters are controlled by the typical control measures of a commercial manufacturing process, including CoA, MBR/MES, and IPCS. The advancement results from the measured actual process parameters, particularly the measured cumulative process parameters, were causally linked by the mechanistic process models. In particular, the developed parameter ranges and associated design space based on the stress-energy model allow for the fundamental control of the underlying physical principles and impacting factors of the size-reduction process for the simple, robust, and sound scientific control of CQA mean particle size. Moreover, the control strategy and associated design space allow for batch size flexibility within the investigated range. This is an often-underestimated benefit for the commercial lifecycle to allow for flexible manufacturing strategies in the downstream processing of nanocrystal suspensions and flexible adaptation to changing demands. The control strategy and associated design space are, strictly speaking, scale-specific and limited to the investigated manufacturing setup. However, the scientific scale-up is supported by the stress-energy model and energy balance and the established ranges of process parameters, including average stress energy, mass specific energy, and suspension outlet temperature, from the grinding chamber, if needed.

## 4. Conclusions

A control strategy and an associated design space were established for a wet media milled nanocrystal suspension at a commercial manufacturing scale. The development was facilitated by a comprehensive QbD approach to identify the relationship between material attributes, process parameters, and quality attributes, and to define their criticality and acceptable ranges in relation to an acceptance criteria range of the CQA mean particle size. A control strategy and design space are built upon mature mechanistic process models, including a stress-energy model for the process–product attribute relationship of the size-reduction process and an energy balance for the temperature control of the suspension in the grinding chamber. This maximizes the active integration of prior knowledge. The design space was developed based on the stress-energy model and related measured cumulative process parameters—mass specific energy and number of turnover cycles—for the physical reasonable control of the impacting factors. We present the process–product attribute relationship with the simple, robust, and sound scientific process control of CQA mean particle size. The control strategy allows for flexible batch sizes within the investigated range to enable different manufacturing strategies in the downstream processing of nanocrystal suspensions and adaptation to changing demands. The executed control strategy considers measures before, during, and after batch manufacturing. This includes a certificate of analysis of input materials, a process control system, master batch records, and executed batch record, as well as an in-process quality control measurement of the CQA mean particle size.

## Figures and Tables

**Table 1:** Overview of material attributes of input components, equipment and process parameters and potential critical quality attributes defined and investigated for the experiments of the manufacturing process at a commercial scale.

Category	Attribute/Parameter	Range Investigated
Material attributes of input components	Drug substance chemical attributes, solid form, residual solvents, water content, impurities, etc.; drug substance concentration; stabilizer type and concentration; water quality and concentration for suspension; water quality and quantity for mechanical seal	Constant
	Drug substance particle size	4.2 μm ≤ x_90,3_ ≤ 7.6 μm
Equipment parameters	Agitator shaft material and geometry; grinding chamber liner material and geometry; sieve cartridge material, geometry and mesh size; pump type and size; pump-tube type and size; vessel stirrer type and size	Constant
	Vessel size, geometry and suspension fill level	Vessel size adapted to batch size with geometrically similar vessel design
Set process parameters	Vessel cooling-liquid volume flow and temperature; mechanical seal cooling-liquid volume flow and temperature; grinding chamber cooling-liquid volume flow; grinding media material (ρ_GM_), diameter (d_GM_) and quantity	Constant
	Vessel stirred speed	Stirrer speed adapted to vessel size and batch size
	Cooling-liquid inlet temperature into grinding chamber jacket ($T_{C,in,i}$)	8–10 °C
	Agitator shaft speed (n_i_)	875–1225 rpm
	Suspension volume flow (${\dot{V}}_{i}$)	5–20 L/min
	Suspension batch size (M)	62–175 kg
Resulting actual process parameters	Agitator shaft tip speed (v_t,i_); suspension mass flow (${\dot{m}}_{i}$); suspension inlet temperature into grinding chamber (T_S,in,i_); suspension outlet temperature from grinding chamber (T_S,out,i_)	Measured process parameters
Resulting cumulative process parameters	Average stress energy ($\bar{SE}$); Mass specific energy (E_M_); number of turnover cycles (N)	Calculated parameters according to Equations (3), (4) and (6)
In-process quality control	Mean particle size (x_PCS_); polydispersity index (PdI)	Measured quality attributes during processing and/or end of manufactured batch

**Table 2:** Overview of experiments, including: (a) Material attribute: Drug substance particle size (x_90,3_), (b) Set process parameters: Suspension batch size (M), agitator shaft speed (n), suspension volume flow ($\dot{V}$), cooling-liquid inlet temperature into grinding chamber jacket (T_C,in_), (c) Resulting process parameters: average suspension outlet temperature from grinding chamber (${\bar{T}}_{S,out}$), mass specific energy (E_M_), average stress energy ($\bar{SE}$), number of turnover cycles (N), and (d) Potential critical quality attributes: Mean particle size (x_PCS_), polydispersity index (PdI).

Experiment No.	Material Attribute	Set Process Parameters				Resulting Process Parameters				Potential Critical Quality Attribute	
	x_90,3_ /μm	M /kg	n /rpm	$\dot{V}$ /L/min	$T_{C,in}$ /°C	${\bar{T}}_{S,out}$ /°C	E_M_/kJ/kg	$\bar{SE}$ /μNm	N /-	x_PCS_ /nm	PdI /-
1	4.8	82	1050	20	9	20	2186	0.88	169	127	0.10
2	4.9	62	1050	20	9	22	2795	0.88	232	119	0.12
3	5.4	62	1225	17	10	27	4007	1.17	213	111	0.13
4	5.0	82	875	13	8	18	2134	0.61	195	123	0.11
5	5.1	82	1050	5	10	23	2134	0.88	57	124	0.11
6	5.1	72	1050	17	8	22	2134	0.88	179	121	0.12
7	5.1	72	1050	19	10	21	2134	0.88	156	128	0.12
8	4.8	155	1050	16	9	24	2134	0.88	79	125	0.12
9	4.4	165	1050	16	9	21	2150	0.88	135	127	0.11
10	4.8	165	1050	16	9	21	2150	0.88	133	132	0.12
11	5.9	165	1050	16	9	21	2140	0.88	136	127	0.11
12	5.7	165	1050	16	9	21	2150	0.87	137	132	0.12
13	6.2	175	1050	16	9	20	640	0.87	36	174	0.11
14	6.6	175	1050	16	9	20	2400	0.87	143	129	0.11
15	7.6	175	1050	16	9	20	1400	0.87	80	144	0.12
16	5.3	175	1050	16	9	21	600	0.88	35	173	0.10
17	4.5	175	1050	16	9	21	2400	0.87	152	129	0.13
18	4.2	175	1050	16	9	20	1400	0.88	87	137	0.12
19	7.6	175	1050	16	9	22	1400	0.87	89	142	0.11
20	5.9	175	1050	16	9	21	1400	0.87	89	138	0.12
21	4.5	175	1050	16	9	21	600	0.87	37	172	0.07
22	5.1	175	1050	16	9	21	2400	0.87	156	127	0.11

**Table 3:** Control strategy and criticality designation for the nanocrystal manufacturing process with exemplary acceptance criteria for mean particle size of 120 nm ≤ x_PCS_ ≤ 180 nm, including control measures by certificate of analysis (CoA), industrial process control system (IPCS), master batch record, related procedures, and manufacturing execution system (MBR/MES) and in-process quality control (IPC).

Category	Attribute/Parameter	Criticality	Control Strategy/Measure
Material attributes of input components	Drug substance chemical attributes, solid form, residual solvents, water content, impurities, etc.; Stabilizer type	No-CMA	CoA
	Drug substance particle size	No-CMA	CoA (x_90,3_ ≤ 7.6 μm)
	Water quality for suspension and mechanical seal	No-CMA	Control procedure in plant
	Drug substance concentration; stabilizer concentration; water concentration for suspension; water quantity for mechanical seal; water quantity for mechanical seal	No-CMA	MBR/MES
Equipment parameters	Agitator shaft material and geometry; grinding chamber liner material and geometry; sieve cartridge material, geometry and mesh size; pump type and size; pump-tube type and size; vessel size and geometry; vessel stirrer type and size	No-CPP	MBR/MES
Set process parameters	Grinding media material (ρ_GM_) and diameter (d_GM_)	No-CPP	CoA, MBR/MES
	Grinding media quantity, respectively fill level in grinding chamber	No-CPP	MBR/MES
	Vessel stirred speed; vessel cooling-liquid volume flow and temperature; mechanical seal cooling-liquid volume flow and temperature; grinding chamber cooling-liquid volume flow	No-CPP	MBR/MES, IPCS
	Cooling-liquid inlet temperature into grinding chamber jacket ($T_{C,in,i}$)	No-CPP	MBR/MES, IPCS (8 °C ≤ T_C,in_ ≤ 13 °C)
	Agitator shaft speed (n_i_)	No-CPP	MBR/MES IPCS (875 rpm ≤ n ≤ 1215 rpm)
	Suspension volume flow (${\dot{V}}_{i}$)	No-CPP	MBR/MES, IPCS (5 L/min ≤ $\dot{V}$ ≤ 20 L/min)
	Suspension batch size (M)	No-CPP	MBR/MES (62 ≤ M ≤ 175 kg)
Resulting actual process parameters	Agitator shaft tip speed (v_t,i_); suspension mass flow (${\dot{m}}_{i}$); suspension inlet temperature into grinding chamber (T_S,in,i_)	No-CPP	IPCS
	Suspension outlet temperature from grinding chamber (T_S,out,i_)	No-CPP	MBR/MES, IPCS (18 °C ≤ T_S,out,i_ ≤ 27 °C)
Resulting cumulative process parameters	Average stress energy ($\bar{SE}$)	No-CPP	MBR/MES, IPCS (0.61 μNm ≤ $\bar{SE}$ ≤ 1.17 μNm)
	Mass specific energy (E_M_)	CPP	MBR/MES, IPCS (see design space in Figure 7)
	Number of turnover cycles (N)	CPP	MBR/MES, IPCS (see design space in Figure 7)
In-process quality control	Mean particle size (x_PCS_)	CQA	IPC (120 nm ≤ x_PCS_ ≤ 180 nm)

## Data Availability

Data beyond the information within the article are unavailable due to intellectual property rights restrictions.
